# 2-(4,5-Diphenyl-2-*p*-tolyl-1*H*-imidazol-1-yl)-3-phenyl­propan-1-ol

**DOI:** 10.1107/S1600536811054766

**Published:** 2012-01-07

**Authors:** Yongmei Xiao, Liangru Yang, Kun He, Jinwei Yuan, Pu Mao

**Affiliations:** a School of Chemistry and Chemical Engineering, Henan University of Technology, Zhengzhou 450001, People’s Republic of China

## Abstract

In the title compound, C_31_H_28_N_2_O, the dihedral angles formed by the imidazole ring with the three aryl substituents are 18.52 (8) and 85.56 (7) and 85.57 (7)°, respectively. In the crystal, mol­ecules are linked by O—H⋯N and C—H⋯O hydrogen bonds into chains parallel to the *a* axis.

## Related literature

For the synthesis and properties of chiral ionic liquids, see: Olivier-Bourbigou *et al.* (2010 ▶); Chen *et al.* (2008 ▶); Mao *et al.* (2010 ▶).
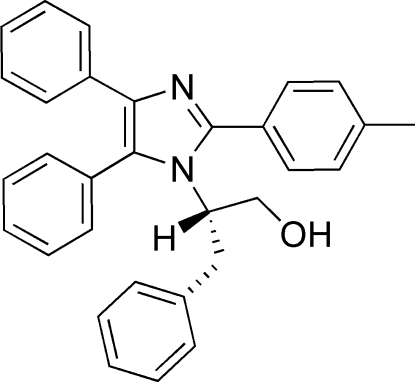


## Experimental

### 

#### Crystal data

C_31_H_28_N_2_O
*M* *_r_* = 444.55
Orthorhombic, 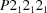

*a* = 9.3413 (7) Å
*b* = 13.7402 (11) Å
*c* = 19.6296 (14) Å
*V* = 2519.5 (3) Å^3^
*Z* = 4
Cu *K*α radiation
μ = 0.55 mm^−1^
*T* = 291 K
0.25 × 0.20 × 0.20 mm

#### Data collection

Agilent Xcalibur Eos Gemini diffractometer
Absorption correction: multi-scan (*CrysAlis PRO*; Agilent, 2011 ▶) *T*_min_ = 0.866, *T*_max_ = 1.000
9302 measured reflections
4441 independent reflections
4007 reflections with *I* > 2σ(*I*)
*R*_int_ = 0.027

#### Refinement

*R*[*F* ^2^ > 2σ(*F* ^2^)] = 0.037
*wR*(*F* ^2^) = 0.098
*S* = 1.03
4441 reflections
313 parameters
H atoms treated by a mixture of independent and constrained refinement
Δρ_max_ = 0.12 e Å^−3^
Δρ_min_ = −0.13 e Å^−3^
Absolute structure: Flack (1983 ▶); 1887 Friedel pairs
Flack parameter: −0.1 (3)

### 

Data collection: *CrysAlis PRO* (Agilent, 2011 ▶); cell refinement: *CrysAlis PRO*; data reduction: *CrysAlis PRO*; program(s) used to solve structure: *SHELXS97* (Sheldrick, 2008 ▶); program(s) used to refine structure: *SHELXL97* (Sheldrick, 2008 ▶); molecular graphics: *OLEX2* (Dolomanov *et al.*, 2009 ▶); software used to prepare material for publication: *OLEX2*.

## Supplementary Material

Crystal structure: contains datablock(s) I, global. DOI: 10.1107/S1600536811054766/rz2683sup1.cif

Structure factors: contains datablock(s) I. DOI: 10.1107/S1600536811054766/rz2683Isup2.hkl

Supplementary material file. DOI: 10.1107/S1600536811054766/rz2683Isup3.cml

Additional supplementary materials:  crystallographic information; 3D view; checkCIF report

## Figures and Tables

**Table 1:** Hydrogen-bond geometry (Å, °)

*D*—H⋯*A*	*D*—H	H⋯*A*	*D*⋯*A*	*D*—H⋯*A*
O1—H1⋯N1^i^	0.82 (3)	2.01 (3)	2.825 (2)	174 (3)
C16—H16⋯O1^ii^	0.93	2.56	3.272 (3)	133

Symmetry codes: (i) 
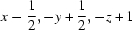
; (ii) 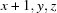.

**Fig. 1.**
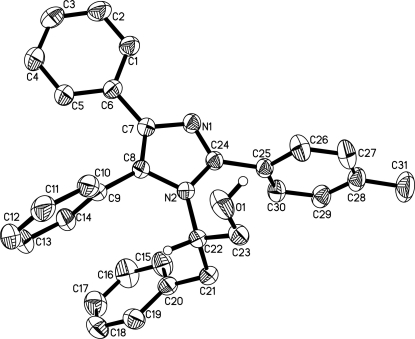
The molecular structure of the title compound showing 30% probability displacement ellipsoids. Hydrogen atoms are omitted for clarity.

## supplementary crystallographic information

### Comment

Ionic liquids (ILs) have received considerable interest in the fields of synthesis, analysis and catalysis due to their unique properties (Olivier-Bourbigou *et al.*, 2010). Chiral ionic liquids (CILs) derived from naturally abundant precursors have also attracted much interest (Chen *et al.*, 2008). Our group is interested in the preparation and application of imidazole derived CILs (Mao *et al.*, 2010), and we observed that the condensation reaction between *L*-phenylalaninol (easily available from *L*-phenylalanine), dibenzoyl, 4-methylbenzaldehyde and ammonium acetate afforded the title compound, a multi-aryl substituted imidazole derivative containing an appended chiral functionality. The chiral C22 carbon atom maintains the *S* configuration observed in *L*-phenylalaninol.

The molecular structure of the title compound is shown in Figure 1. As expected, the imidazole core (N1/C7/C8/N2/C24) is essentially planar. The dihedral angles formed by the imidazole ring with the three aryl substituents are 18.52 (8) (C1–C6), 85.56 (7) (C9–C14) and 85.57 (7)° (C25–C30), respectively. In the crystal structure, molecules are linked by O—H···N and C—H···O hydrogen bonds (Table 1) into chains parallel to the *a* axis.

### Experimental

To a solution of *L*-phenylalaninol (15.1 g, 0.1 mol) in MeOH (50 ml) in an ice-bath, a molar equivalent of dibenzoyl, 4-methylbenzaldehyde and ammonium acetate were added. The mixture was kept stirring in the ice-bath until all the solids were dissolved before being heated to 60°C for 5 h. The mixture was then cooled to room temperature and the solvent was removed by evaporation. The residue was washed with H_2_O to obtain the crude product. Crystallization of the crude product in EtOH afforded colourless crystals of the title compound.

### Refinement

The hydroxyl H atom was located in a difference Fourier map and refined freely. All other H atoms were placed in calculated positions with C—H = 0.93–0.98 Å and refined as riding, with *U*_iso_(H) = 1.2*U*_eq_(C) or 1.5*U*_eq_(C) for methyl H atoms.

### Crystal data

**Table:**

C_31_H_28_N_2_O	*F*(000) = 944
*M**_r_* = 444.55	*D*_x_ = 1.172 Mg m^−^^3^
Orthorhombic, *P*2_1_2_1_2_1_	Cu *K*α radiation, λ = 1.5418 Å
Hall symbol: P 2ac 2ab	Cell parameters from 3569 reflections
*a* = 9.3413 (7) Å	θ = 3.2–67.0°
*b* = 13.7402 (11) Å	µ = 0.55 mm^−^^1^
*c* = 19.6296 (14) Å	*T* = 291 K
*V* = 2519.5 (3) Å^3^	Prismatic, colourless
*Z* = 4	0.25 × 0.20 × 0.20 mm

### Data collection

**Table:**

Agilent Xcalibur Eos Gemini diffractometer	4441 independent reflections
Radiation source: Enhance (Cu) X-ray Source	4007 reflections with *I* > 2σ(*I*)
graphite	*R*_int_ = 0.027
Detector resolution: 16.2312 pixels mm^-1^	θ_max_ = 66.9°, θ_min_ = 3.9°
ω scans	*h* = −9→11
Absorption correction: multi-scan (*CrysAlis PRO*; Agilent, 2011)	*k* = −16→13
*T*_min_ = 0.866, *T*_max_ = 1.000	*l* = −21→23
9302 measured reflections	

### Refinement

**Table:**

Refinement on *F*^2^	Hydrogen site location: inferred from neighbouring sites
Least-squares matrix: full	H atoms treated by a mixture of independent and constrained refinement
*R*[*F*^2^ > 2σ(*F*^2^)] = 0.037	*w* = 1/[σ^2^(*F*_o_^2^) + (0.0481*P*)^2^ + 0.1385*P*] where *P* = (*F*_o_^2^ + 2*F*_c_^2^)/3
*wR*(*F*^2^) = 0.098	(Δ/σ)_max_ < 0.001
*S* = 1.03	Δρ_max_ = 0.12 e Å^−^^3^
4441 reflections	Δρ_min_ = −0.13 e Å^−^^3^
313 parameters	Extinction correction: *SHELXL97* (Sheldrick, 2008), Fc^*^=kFc[1+0.001xFc^2^λ^3^/sin(2θ)]^-1/4^
0 restraints	Extinction coefficient: 0.0029 (2)
Primary atom site location: structure-invariant direct methods	Absolute structure: Flack (1983); 1887 Friedel pairs
Secondary atom site location: difference Fourier map	Flack parameter: −0.1 (3)

### Special details

**Table:**

Geometry. All e.s.d.'s (except the e.s.d. in the dihedral angle between two l.s. planes) are estimated using the full covariance matrix. The cell e.s.d.'s are taken into account individually in the estimation of e.s.d.'s in distances, angles and torsion angles; correlations between e.s.d.'s in cell parameters are only used when they are defined by crystal symmetry. An approximate (isotropic) treatment of cell e.s.d.'s is used for estimating e.s.d.'s involving l.s. planes.
Refinement. Refinement of *F*^2^ against ALL reflections. The weighted *R*-factor *wR* and goodness of fit *S* are based on *F*^2^, conventional *R*-factors *R* are based on *F*, with *F* set to zero for negative *F*^2^. The threshold expression of *F*^2^ > σ(*F*^2^) is used only for calculating *R*-factors(gt) *etc*. and is not relevant to the choice of reflections for refinement. *R*-factors based on *F*^2^ are statistically about twice as large as those based on *F*, and *R*- factors based on ALL data will be even larger.

### Fractional atomic coordinates and isotropic or equivalent isotropic displacement parameters (Å^2^)

**Table:**

	*x*	*y*	*z*	*U*_iso_*/*U*_eq_	
O1	0.03250 (17)	0.28449 (11)	0.37156 (10)	0.0793 (5)	
N1	0.38409 (17)	0.31434 (10)	0.52389 (7)	0.0523 (3)	
N2	0.32181 (16)	0.32519 (10)	0.41559 (7)	0.0479 (3)	
C1	0.3733 (2)	0.45666 (14)	0.63052 (9)	0.0622 (5)	
H1A	0.3616	0.3912	0.6413	0.075*	
C2	0.3881 (3)	0.52437 (18)	0.68230 (11)	0.0786 (6)	
H2	0.3855	0.5040	0.7275	0.094*	
C3	0.4063 (4)	0.62017 (19)	0.66770 (12)	0.0932 (8)	
H3	0.4181	0.6652	0.7026	0.112*	
C4	0.4069 (4)	0.64981 (17)	0.60113 (13)	0.1027 (10)	
H4	0.4174	0.7155	0.5909	0.123*	
C5	0.3922 (3)	0.58302 (15)	0.54886 (11)	0.0797 (7)	
H5	0.3933	0.6043	0.5039	0.096*	
C6	0.3757 (2)	0.48512 (13)	0.56267 (9)	0.0547 (4)	
C7	0.3616 (2)	0.41103 (12)	0.50849 (8)	0.0497 (4)	
C8	0.32230 (19)	0.41909 (11)	0.44146 (8)	0.0474 (4)	
C9	0.2811 (2)	0.50422 (12)	0.39900 (8)	0.0518 (4)	
C10	0.1384 (3)	0.52990 (14)	0.39201 (10)	0.0646 (5)	
H10	0.0678	0.4931	0.4133	0.077*	
C11	0.1006 (3)	0.61040 (17)	0.35333 (12)	0.0844 (7)	
H11	0.0046	0.6273	0.3489	0.101*	
C12	0.2033 (4)	0.66518 (16)	0.32168 (13)	0.0948 (9)	
H12	0.1776	0.7197	0.2964	0.114*	
C13	0.3428 (4)	0.63930 (17)	0.32753 (13)	0.0941 (9)	
H13	0.4124	0.6759	0.3054	0.113*	
C14	0.3837 (3)	0.55919 (14)	0.36594 (11)	0.0719 (6)	
H14	0.4799	0.5425	0.3694	0.086*	
C15	0.6439 (3)	0.33594 (17)	0.33126 (13)	0.0782 (6)	
H15	0.6546	0.2910	0.3663	0.094*	
C16	0.7497 (3)	0.4045 (2)	0.32048 (17)	0.0972 (8)	
H16	0.8298	0.4058	0.3486	0.117*	
C17	0.7373 (3)	0.4703 (2)	0.26865 (16)	0.0901 (7)	
H17	0.8090	0.5160	0.2611	0.108*	
C18	0.6186 (3)	0.46844 (18)	0.22809 (12)	0.0836 (7)	
H18	0.6088	0.5135	0.1931	0.100*	
C19	0.5128 (3)	0.39962 (16)	0.23896 (10)	0.0694 (5)	
H19	0.4333	0.3984	0.2105	0.083*	
C20	0.5231 (2)	0.33255 (13)	0.29131 (9)	0.0551 (4)	
C21	0.4054 (2)	0.25978 (13)	0.30306 (9)	0.0559 (4)	
H21A	0.4449	0.2037	0.3265	0.067*	
H21B	0.3697	0.2378	0.2593	0.067*	
C22	0.2799 (2)	0.29999 (12)	0.34487 (8)	0.0482 (4)	
H22	0.2516	0.3611	0.3230	0.058*	
C23	0.1490 (2)	0.23495 (13)	0.34372 (9)	0.0562 (4)	
H23A	0.1276	0.2161	0.2972	0.067*	
H23B	0.1675	0.1763	0.3698	0.067*	
C24	0.35972 (19)	0.26487 (12)	0.46788 (8)	0.0478 (4)	
C25	0.3693 (2)	0.15676 (12)	0.46470 (8)	0.0505 (4)	
C26	0.2543 (3)	0.10109 (16)	0.48526 (13)	0.0757 (6)	
H26	0.1712	0.1313	0.5004	0.091*	
C27	0.2621 (3)	0.00056 (17)	0.48343 (15)	0.0865 (7)	
H27	0.1833	−0.0358	0.4973	0.104*	
C28	0.3829 (3)	−0.04669 (14)	0.46169 (11)	0.0725 (6)	
C29	0.4981 (3)	0.00908 (15)	0.44320 (11)	0.0697 (5)	
H29	0.5818	−0.0214	0.4290	0.084*	
C30	0.4930 (2)	0.10967 (13)	0.44520 (11)	0.0611 (5)	
H30	0.5735	0.1457	0.4333	0.073*	
C31	0.3906 (5)	−0.15668 (17)	0.45750 (18)	0.1156 (12)	
H31A	0.3258	−0.1847	0.4900	0.173*	
H31B	0.4864	−0.1777	0.4674	0.173*	
H31C	0.3645	−0.1774	0.4125	0.173*	
H1	−0.008 (3)	0.2522 (19)	0.4008 (15)	0.090 (9)*	

### Atomic displacement parameters (Å^2^)

**Table:**

	*U*^11^	*U*^22^	*U*^33^	*U*^12^	*U*^13^	*U*^23^
O1	0.0651 (9)	0.0671 (9)	0.1056 (12)	0.0056 (7)	0.0218 (8)	0.0281 (9)
N1	0.0603 (8)	0.0461 (7)	0.0505 (7)	0.0012 (6)	−0.0088 (6)	0.0028 (6)
N2	0.0599 (8)	0.0403 (6)	0.0434 (7)	0.0029 (6)	0.0022 (6)	0.0007 (5)
C1	0.0732 (12)	0.0594 (10)	0.0540 (9)	0.0054 (10)	−0.0049 (9)	−0.0026 (7)
C2	0.1002 (17)	0.0851 (15)	0.0506 (10)	0.0051 (13)	−0.0054 (11)	−0.0118 (10)
C3	0.135 (2)	0.0790 (15)	0.0656 (13)	−0.0143 (16)	0.0045 (14)	−0.0287 (11)
C4	0.173 (3)	0.0568 (12)	0.0780 (15)	−0.0268 (16)	0.0106 (17)	−0.0160 (11)
C5	0.129 (2)	0.0531 (10)	0.0574 (10)	−0.0127 (12)	0.0033 (12)	−0.0049 (8)
C6	0.0599 (10)	0.0525 (9)	0.0517 (9)	0.0002 (8)	−0.0003 (8)	−0.0063 (7)
C7	0.0563 (9)	0.0440 (8)	0.0489 (8)	−0.0007 (7)	0.0002 (7)	−0.0013 (6)
C8	0.0543 (9)	0.0406 (7)	0.0472 (8)	0.0015 (7)	0.0059 (7)	−0.0005 (6)
C9	0.0729 (11)	0.0409 (8)	0.0417 (8)	0.0063 (8)	0.0082 (7)	−0.0005 (6)
C10	0.0804 (13)	0.0544 (10)	0.0588 (10)	0.0156 (10)	0.0056 (9)	0.0002 (8)
C11	0.116 (2)	0.0647 (12)	0.0726 (13)	0.0359 (14)	−0.0086 (13)	−0.0029 (11)
C12	0.162 (3)	0.0499 (11)	0.0730 (14)	0.0280 (16)	0.0052 (16)	0.0124 (10)
C13	0.147 (3)	0.0552 (12)	0.0799 (15)	−0.0041 (15)	0.0280 (17)	0.0176 (10)
C14	0.0925 (16)	0.0530 (10)	0.0701 (12)	−0.0028 (10)	0.0186 (12)	0.0086 (9)
C15	0.0719 (13)	0.0700 (12)	0.0927 (15)	0.0040 (11)	−0.0099 (12)	0.0185 (11)
C16	0.0627 (14)	0.0957 (18)	0.133 (2)	−0.0085 (14)	−0.0198 (15)	0.0150 (17)
C17	0.0751 (15)	0.0857 (16)	0.109 (2)	−0.0166 (13)	0.0163 (14)	0.0086 (15)
C18	0.0989 (18)	0.0788 (14)	0.0731 (13)	−0.0138 (13)	0.0097 (13)	0.0166 (11)
C19	0.0784 (13)	0.0721 (12)	0.0577 (10)	−0.0050 (11)	−0.0024 (10)	0.0057 (9)
C20	0.0588 (10)	0.0537 (9)	0.0528 (10)	0.0077 (8)	0.0093 (8)	−0.0044 (7)
C21	0.0692 (11)	0.0478 (9)	0.0507 (9)	0.0043 (8)	0.0052 (8)	−0.0058 (7)
C22	0.0614 (10)	0.0412 (7)	0.0422 (8)	0.0013 (7)	0.0021 (7)	0.0012 (6)
C23	0.0647 (11)	0.0535 (9)	0.0505 (9)	−0.0047 (8)	−0.0015 (8)	0.0071 (7)
C24	0.0526 (9)	0.0429 (8)	0.0479 (8)	0.0019 (7)	−0.0032 (7)	0.0025 (6)
C25	0.0601 (10)	0.0430 (8)	0.0484 (8)	−0.0011 (8)	−0.0097 (8)	0.0038 (6)
C26	0.0677 (13)	0.0595 (11)	0.0997 (17)	−0.0002 (10)	0.0061 (12)	0.0179 (11)
C27	0.0852 (16)	0.0605 (12)	0.1136 (19)	−0.0212 (12)	−0.0098 (14)	0.0231 (12)
C28	0.1010 (17)	0.0450 (9)	0.0716 (12)	−0.0019 (11)	−0.0277 (12)	0.0060 (8)
C29	0.0806 (14)	0.0497 (10)	0.0788 (13)	0.0136 (10)	−0.0095 (11)	0.0008 (9)
C30	0.0617 (11)	0.0486 (9)	0.0731 (11)	0.0009 (9)	−0.0096 (9)	0.0038 (8)
C31	0.167 (3)	0.0480 (12)	0.131 (3)	−0.0091 (17)	−0.024 (2)	0.0048 (13)

### Geometric parameters (Å, °)

**Table:**

O1—C23	1.395 (3)	C15—C16	1.382 (4)
O1—H1	0.82 (3)	C15—C20	1.375 (3)
N1—C7	1.379 (2)	C16—H16	0.9300
N1—C24	1.312 (2)	C16—C17	1.366 (4)
N2—C8	1.387 (2)	C17—H17	0.9300
N2—C22	1.483 (2)	C17—C18	1.365 (4)
N2—C24	1.366 (2)	C18—H18	0.9300
C1—H1A	0.9300	C18—C19	1.384 (3)
C1—C2	1.385 (3)	C19—H19	0.9300
C1—C6	1.388 (3)	C19—C20	1.383 (3)
C2—H2	0.9300	C20—C21	1.503 (3)
C2—C3	1.358 (4)	C21—H21A	0.9700
C3—H3	0.9300	C21—H21B	0.9700
C3—C4	1.369 (4)	C21—C22	1.534 (2)
C4—H4	0.9300	C22—H22	0.9800
C4—C5	1.383 (3)	C22—C23	1.515 (3)
C5—H5	0.9300	C23—H23A	0.9700
C5—C6	1.381 (3)	C23—H23B	0.9700
C6—C7	1.478 (2)	C24—C25	1.489 (2)
C7—C8	1.371 (2)	C25—C26	1.379 (3)
C8—C9	1.487 (2)	C25—C30	1.379 (3)
C9—C10	1.386 (3)	C26—H26	0.9300
C9—C14	1.382 (3)	C26—C27	1.384 (3)
C10—H10	0.9300	C27—H27	0.9300
C10—C11	1.387 (3)	C27—C28	1.370 (4)
C11—H11	0.9300	C28—C29	1.370 (4)
C11—C12	1.368 (4)	C28—C31	1.515 (3)
C12—H12	0.9300	C29—H29	0.9300
C12—C13	1.355 (5)	C29—C30	1.384 (3)
C13—H13	0.9300	C30—H30	0.9300
C13—C14	1.388 (3)	C31—H31A	0.9600
C14—H14	0.9300	C31—H31B	0.9600
C15—H15	0.9300	C31—H31C	0.9600
			
C23—O1—H1	111.8 (19)	C18—C17—H17	120.3
C24—N1—C7	106.80 (13)	C17—C18—H18	119.9
C8—N2—C22	124.11 (13)	C17—C18—C19	120.2 (2)
C24—N2—C8	106.77 (13)	C19—C18—H18	119.9
C24—N2—C22	129.06 (13)	C18—C19—H19	119.3
C2—C1—H1A	119.6	C20—C19—C18	121.3 (2)
C2—C1—C6	120.89 (19)	C20—C19—H19	119.3
C6—C1—H1A	119.6	C15—C20—C19	117.2 (2)
C1—C2—H2	119.7	C15—C20—C21	122.33 (18)
C3—C2—C1	120.6 (2)	C19—C20—C21	120.43 (18)
C3—C2—H2	119.7	C20—C21—H21A	108.8
C2—C3—H3	120.3	C20—C21—H21B	108.8
C2—C3—C4	119.4 (2)	C20—C21—C22	113.64 (14)
C4—C3—H3	120.3	H21A—C21—H21B	107.7
C3—C4—H4	119.7	C22—C21—H21A	108.8
C3—C4—C5	120.7 (2)	C22—C21—H21B	108.8
C5—C4—H4	119.7	N2—C22—C21	112.53 (15)
C4—C5—H5	119.6	N2—C22—H22	106.3
C6—C5—C4	120.8 (2)	N2—C22—C23	111.38 (13)
C6—C5—H5	119.6	C21—C22—H22	106.3
C1—C6—C7	119.66 (16)	C23—C22—C21	113.37 (14)
C5—C6—C1	117.68 (17)	C23—C22—H22	106.3
C5—C6—C7	122.65 (17)	O1—C23—C22	109.65 (15)
N1—C7—C6	119.49 (14)	O1—C23—H23A	109.7
C8—C7—N1	109.22 (14)	O1—C23—H23B	109.7
C8—C7—C6	131.23 (15)	C22—C23—H23A	109.7
N2—C8—C9	121.73 (14)	C22—C23—H23B	109.7
C7—C8—N2	106.10 (14)	H23A—C23—H23B	108.2
C7—C8—C9	132.14 (15)	N1—C24—N2	111.12 (14)
C10—C9—C8	120.32 (17)	N1—C24—C25	122.76 (14)
C14—C9—C8	120.90 (18)	N2—C24—C25	126.10 (14)
C14—C9—C10	118.77 (18)	C26—C25—C24	119.62 (18)
C9—C10—H10	119.9	C30—C25—C24	122.01 (17)
C9—C10—C11	120.1 (2)	C30—C25—C26	118.28 (17)
C11—C10—H10	119.9	C25—C26—H26	119.8
C10—C11—H11	119.7	C25—C26—C27	120.4 (2)
C12—C11—C10	120.6 (3)	C27—C26—H26	119.8
C12—C11—H11	119.7	C26—C27—H27	119.2
C11—C12—H12	120.3	C28—C27—C26	121.6 (2)
C13—C12—C11	119.4 (2)	C28—C27—H27	119.2
C13—C12—H12	120.3	C27—C28—C29	117.69 (18)
C12—C13—H13	119.4	C27—C28—C31	121.9 (3)
C12—C13—C14	121.3 (3)	C29—C28—C31	120.4 (3)
C14—C13—H13	119.4	C28—C29—H29	119.2
C9—C14—C13	119.8 (3)	C28—C29—C30	121.6 (2)
C9—C14—H14	120.1	C30—C29—H29	119.2
C13—C14—H14	120.1	C25—C30—C29	120.4 (2)
C16—C15—H15	119.2	C25—C30—H30	119.8
C20—C15—H15	119.2	C29—C30—H30	119.8
C20—C15—C16	121.5 (2)	C28—C31—H31A	109.5
C15—C16—H16	119.9	C28—C31—H31B	109.5
C17—C16—C15	120.3 (2)	C28—C31—H31C	109.5
C17—C16—H16	119.9	H31A—C31—H31B	109.5
C16—C17—H17	120.3	H31A—C31—H31C	109.5
C18—C17—C16	119.4 (2)	H31B—C31—H31C	109.5
			
N1—C7—C8—N2	−0.31 (19)	C11—C12—C13—C14	−1.1 (4)
N1—C7—C8—C9	177.69 (18)	C12—C13—C14—C9	0.1 (4)
N1—C24—C25—C26	82.9 (2)	C14—C9—C10—C11	−1.1 (3)
N1—C24—C25—C30	−93.5 (2)	C15—C16—C17—C18	0.7 (5)
N2—C8—C9—C10	84.7 (2)	C15—C20—C21—C22	97.1 (2)
N2—C8—C9—C14	−95.4 (2)	C16—C15—C20—C19	1.1 (4)
N2—C22—C23—O1	63.07 (19)	C16—C15—C20—C21	−178.8 (2)
N2—C24—C25—C26	−95.2 (2)	C16—C17—C18—C19	−0.8 (4)
N2—C24—C25—C30	88.4 (2)	C17—C18—C19—C20	1.1 (4)
C1—C2—C3—C4	1.4 (5)	C18—C19—C20—C15	−1.2 (3)
C1—C6—C7—N1	−17.1 (3)	C18—C19—C20—C21	178.7 (2)
C1—C6—C7—C8	159.8 (2)	C19—C20—C21—C22	−82.8 (2)
C2—C1—C6—C5	−0.5 (3)	C20—C15—C16—C17	−0.9 (5)
C2—C1—C6—C7	179.2 (2)	C20—C21—C22—N2	−64.97 (19)
C2—C3—C4—C5	−1.3 (6)	C20—C21—C22—C23	167.51 (15)
C3—C4—C5—C6	0.3 (5)	C21—C22—C23—O1	−168.81 (14)
C4—C5—C6—C1	0.5 (4)	C22—N2—C8—C7	177.67 (16)
C4—C5—C6—C7	−179.1 (3)	C22—N2—C8—C9	−0.6 (3)
C5—C6—C7—N1	162.6 (2)	C22—N2—C24—N1	−177.41 (16)
C5—C6—C7—C8	−20.5 (3)	C22—N2—C24—C25	0.9 (3)
C6—C1—C2—C3	−0.5 (4)	C24—N1—C7—C6	177.73 (16)
C6—C7—C8—N2	−177.50 (18)	C24—N1—C7—C8	0.2 (2)
C6—C7—C8—C9	0.5 (3)	C24—N2—C8—C7	0.34 (19)
C7—N1—C24—N2	0.1 (2)	C24—N2—C8—C9	−177.92 (16)
C7—N1—C24—C25	−178.29 (17)	C24—N2—C22—C21	−69.1 (2)
C7—C8—C9—C10	−93.1 (3)	C24—N2—C22—C23	59.5 (2)
C7—C8—C9—C14	86.8 (2)	C24—C25—C26—C27	−179.2 (2)
C8—N2—C22—C21	114.21 (17)	C24—C25—C30—C29	179.66 (18)
C8—N2—C22—C23	−117.21 (17)	C25—C26—C27—C28	0.3 (4)
C8—N2—C24—N1	−0.3 (2)	C26—C25—C30—C29	3.2 (3)
C8—N2—C24—C25	178.03 (17)	C26—C27—C28—C29	1.6 (4)
C8—C9—C10—C11	178.86 (17)	C26—C27—C28—C31	−178.1 (3)
C8—C9—C14—C13	−178.9 (2)	C27—C28—C29—C30	−1.0 (3)
C9—C10—C11—C12	0.1 (3)	C28—C29—C30—C25	−1.4 (3)
C10—C9—C14—C13	1.0 (3)	C30—C25—C26—C27	−2.7 (3)
C10—C11—C12—C13	1.0 (4)	C31—C28—C29—C30	178.7 (2)

### Hydrogen-bond geometry (Å, °)

**Table:**

*D*—H···*A*	*D*—H	H···*A*	*D*···*A*	*D*—H···*A*
O1—H1···N1^i^	0.82 (3)	2.01 (3)	2.825 (2)	174 (3)
C16—H16···O1^ii^	0.93	2.56	3.272 (3)	133

Symmetry codes: (i) *x*−1/2, −*y*+1/2, −*z*+1; (ii) *x*+1, *y*, *z*.

## References

[bb1] Agilent (2011). *CrysAlis PRO* Agilent Technologies Ltd, Yarnton, England.

[bb2] Chen, X., Li, X., Hu, A. & Wang, F. (2008). *Tetrahedron Asymmetry*, **19**, 1–14.

[bb3] Dolomanov, O. V., Bourhis, L. J., Gildea, R. J., Howard, J. A. K. & Puschmann, H. (2009). *J. Appl. Cryst.* **42**, 339–341.

[bb4] Flack, H. D. (1983). *Acta Cryst.* A**39**, 876–881.

[bb5] Mao, P., Cai, Y., Xiao, Y., Yang, L., Xue, Y. & Song, M. (2010). *Phosphorus Sulfur Silicon Relat. Elem.* **185**, 2418–2425.

[bb6] Olivier-Bourbigou, H., Magna, L. & Morvan, D. (2010). *Appl. Catal. A*, **373**, 1–56.

[bb7] Sheldrick, G. M. (2008). *Acta Cryst.* A**64**, 112–122. 10.1107/S0108767307043930 18156677

